# Attributions for extreme weather events: science and the people

**DOI:** 10.1007/s10584-022-03443-7

**Published:** 2022-10-14

**Authors:** John McClure, Ilan Noy, Yoshi Kashima, Taciano L. Milfont

**Affiliations:** 1 Victoria University of Wellington, PO Box 600, Wellington, New Zealand; 2 University of Melbourne, Melbourne, Australia; 3 University of Waikato, Tauranga, New Zealand

**Keywords:** Climate change, Extreme event attribution, Science communication, Attribution theory, Probable and sufficient causes

## Abstract

Both climate scientists and non-scientists (laypeople) attribute extreme weather events to various influences. Laypeople’s attributions for these events are important as these attributions likely influence their views and actions about climate change and extreme events. Research has examined laypeople’s attribution scepticism about climate change in general; however, few climate scientists are familiar with the processes underpinning laypeople’s attributions for individual extreme events. Understanding these lay attributions is important for scientists to communicate their findings to the public. Following a brief summary of the way climate scientists calculate attributions for extreme weather events, we focus on cognitive and motivational processes that underlie laypeople’s attributions for specific events. These include a tendency to prefer single-cause rather than multiple-cause explanations, a discounting of whether possible causes covary with extreme events, a preference for sufficient causes over probabilities, applying prevailing causal narratives, and the influence of motivational factors. For climate scientists and communicators who wish to inform the public about the role of climate change in extreme weather events, these patterns suggest several strategies to explain scientists’ attributions for these events and enhance public engagement with climate change. These strategies include showing more explicitly that extreme weather events reflect multiple causal influences, that climate change is a mechanism that covaries with these events and increases the probability and intensity of many of these events, that human emissions contributing to climate change are controllable, and that misleading communications about weather attributions reflect motivated interests rather than good evidence.

## Introduction

Both climate scientists and non-scientists (laypeople) attribute extreme weather events to causal influences. More specifically, both groups make inferences with respect to the contribution of anthropogenic climate change and natural processes to these events. Laypeople’s attributions for extreme weather events are important as they may shape laypeople’s decisions whether to mitigate climate change by reducing their greenhouse gas (GHG) emissions, and how to adapt to the potential destructive impacts of extreme weather events by moving house or changing their lives and livelihoods. These lay attributions are also important in the political sphere, as extreme events have significant costs and people’s attributions for these events could determine who ends up bearing the costs associated with the changing climate. The assignment of ultimate pecuniary liability has ethical, political, and legal components, and the public’s view on the cause and ‘who or what is to blame’ can shape all of these dimensions directly and indirectly through their influence on the political process. People’s judgments of blame reflect their personal beliefs as well as their causal attributions for events (Alicke 2000).

Thus, laypeople’s attributions for extreme weather events (hereafter EWEs) are likely to mediate between these events and people’s climate change engagement. For example, a community may need to decide whether to relocate after a damaging storm (a managed retreat) and who should be paying for it, and their decision is likely to reflect their own attribution for the event, rather than the rigorous scientific view about it. Recent analysis has considered ways in which weather forecasting service providers should approach the task of EWE attribution (Stone et al. 2021). However, without an understanding of laypeople’s EWE attribution rather than climate scientists’, it may be difficult to create appropriate political and individual responses to climate change.

Social science research has shown that laypeople’s views about climate change intertwine with their political and religious beliefs, and that laypeople live in information silos where they exchange messages that reinforce their beliefs (Moser 2016). Other research has shown how biases in laypeople’s judgment and decision-making such as the availability bias affect their perceptions of climate change (e.g. Fischhoff 2021). For example, laypeople often misunderstand confidence intervals unless these are expressed clearly in communications that avoid phrases like ‘at least’ to indicate a lower boundary of the interval (van Oldenborgh et al. 2021). Research has also shown that the framing of risk communications shapes people’s perception of events. As noted by Lidskog and Sjödin (2015), ‘The framing of an event establishes whether it is seen as hazardous or harmless, natural or man-made (sic), preventable or inevitable, and …. whether action is needed and, if so, what kind of action and by whom’ (p. 154).

An example is framing climate change as a climate crisis or emergency rather than global warming (the latter can sound benign in colder climates). Climate science communications can also frame the perceived costs of adaptive actions as forgone benefits, which enhances laypeople’s willingness to act (Hurlstone et al. 2014). Other strategies include climate myth inoculation (Cook 2019; Farrell et al. 2019) and communicating the co-benefits of mitigation actions to climate change deniers (Bain et al. 2016). All these lines of research are extremely useful; however, they do not draw on research on laypeople’s normal patterns of attribution.

More relevant to this attribution question is research on laypeople’s scepticism about climate change. This work has shown that a key constituent of this scepticism is the belief that the changing climate in general is not caused by human activity (Hornsey et al. 2018; Poortinga et al. 2019). These sceptics include deniers, who see the changing climate as associated solely with natural causes rather than human activity, and doubters, who are unsure of the cause(s) of climate change (Haltinner and Sarathchandra 2021; Rahmstorf 2004). This scepticism about climate change relates to laypeople’s political ideology, environmental attitudes, demographic variables, and conspiracy ideation (Hornsey et al. 2016, 2018; Milfont et al. 2015; Poortinga et al. 2019). This research is valuable in revealing citizens’ views about climate change in general, but it does not reveal the cognitive and motivational processes that underpin laypeople’s attributions for specific extreme events such as the 2022 summer drought in continental Europe, or recent fires in California. This omission limits the tools that science communicators can use to address laypeople’s attributions for specific weather events.

The key issue is that few researchers have examined how laypeople’s attributions for specific events might shape their understanding of EWEs (for partial exceptions, see Hannart et al. 2016; Kumagai et al. 2004). In response to this important gap in the literature, here, we describe common patterns in lay attributions for events. As there is little research on lay attributions for EWEs, our discussion draws on lay attributions for events in general. An understanding of these lay attributions may enable climate scientists and communicators to communicate their own attributions for EWEs more persuasively. This strategy may enhance citizen’s engagement with debates about climate change mitigation and adaptation, and may prevent them from either losing interest or losing hope (Bostrom et al. 2013; McClure 2017). Before concentrating on our primary topic of patterns of lay attributions for events in general, we briefly summarise climate scientists’ attributions for EWEs.

## Climate scientists’ attributions for weather events

Since the first Intergovernmental Panel on Climate Change (IPCC) Report in 1990, climate scientists have been claiming, with increasing confidence, that the intensification of GHG emissions influences the frequency and intensity of hurricanes, heatwaves, droughts, and other EWEs. Initially, scientists lacked adequate tools to estimate the role of climate change in the occurrence of specific events (e.g. a hurricane hitting the coast near Houston, Texas). Only recently, following the approach described in Allen (2003) and Stott et al. (2004), have climate scientists been able to quantify climate change contribution to specific weather events, described as the science of extreme event attribution (EEA) by the National Academies of Sciences, Engineering and Medicine, hereafter NASEM (2016). Early work applied this strategy to the UK flood of 2000 and the European heatwaves of 2003 and 2010 (Otto et al. 2012; Pall et al. 2011; Stott et al. 2004). Herring et al. (2020) document 2018 events, and more recent studies investigated events such as the Pacific Northwest heatwave of 2021 and the June 2022 European heatwave. Climate scientists can now show which of these specific events were made more or less likely or severe by anthropogenic climate change (Osaka and Bellamy 2020; Stott et al. 2016).

The science of EEA aims to distinguish the contribution of climate change to an extreme event from the normally prevailing climate and the random variability of the weather. The methods that climate scientists use for this research are well described elsewhere (Hannart et al. 2016; James et al. 2019; Lidskog and Sjödin 2015; NASEM 2016; Stott et al. 2016), and are only mentioned here, since our focus is on lay attributions. In brief, climate scientists use two main sources of information to understand EWEs (NASEM 2016). The first is the observed historical record, which can show whether the probability or intensity of such events has changed significantly over time. However, rarely is this record long and accurate enough for robust conclusions about the role of climate change in these events. This role, however, can be inferred by using a second source of information, namely, climate models that simulate extreme events and study the effects of changing inputs into the model (e.g. changing the levels of GHGs). These modelling studies calculate the probability that a specific weather event would occur with and without these changes, to make inferences on the role of climate change (Stott et al. 2016). This modelling is more accurate for events more directly affected by climate change (e.g. heatwaves) than events with more contributing factors (e.g. the natural and human triggers in wildfires, besides ambient heat) (Harrington et al. 2022). An alternative method to calculating probabilities uses a ‘storyline’ approach to extreme events that examines the role of each factor contributing to each event as the event unfolded, much like an accident investigation (Shepherd 2016; Shepherd et al. 2018).

The higher frequency of EWEs in recent years reinforces the importance of the research on these events, which can inform both public perceptions and government action with respect to climate change mitigation and adaptation (James et al. 2019). Indeed, evidence on how human actions are changing the climate system is important in relation to lay attributions because it is the linchpin on which public policies dealing with compensation, adaptation, and mitigation depend (Harrington et al. 2022). Scientific information about EEA is also useful to the insurance industry, to litigators who take action against large GHG emitters by quantifying attributable damages, and to policy makers developing strategies for climate adaptation and disaster risk reduction (Harrington et al. 2022; Stott et al. 2016). The following sections clarify ways in which communications about EWEs could be enhanced by understanding how laypeople react to these scientific messages and derive their own attributions for EWEs.

## Communicating EEA: the issue of variability in scientific messages

One key aim of EEA is to provide a scientific perspective on local extreme events that communities can use to form mitigation and adaptation policies (Allen 2003). A challenge with this goal is that different climate models may arrive at different calculations about the changing likelihood of EWEs (NASEM 2016; Osaka and Bellamy 2020). Because of this variability in the calculated probabilities (the ‘Fraction of Attributable Risk’) due to differences in climate models, climate scientists sometimes disagree on whether a given extreme event can be attributed to climate change. This variability may affect laypeople’s and policy makers’ understanding and engagement with climate attribution science. If the consensus among climate scientists about climate change is a key factor that has convinced laypeople of the reality of climate change, a lack of consensus about EEA within the science community may undermine laypeople’s and policy makers’ confidence in these attributions and their engagement with mitigation and adaptation actions (Osaka and Bellamy 2020; Stott et al. 2016). This is because many laypeople wrongly believe that scientists must agree on every point for an issue to have scientific merit (Bucchi and Trench 2021).

To illustrate, in research on the effects of communicating EEA to laypeople, Osaka and Bellamy (2020) examined the effects of reporting the findings of EEA science to two focus groups, one of laypeople and one of stakeholders including policy makers and NGO representatives. Using the 2011–2017 California drought as an exemplar, the researchers presented several models of EEA using different formulae employed by climate scientists. Many participants in both groups viewed climate change as a secondary factor in the California drought, but others had not heard of EEA or doubted that scientists could link the drought to anthropogenic climate change. Even participants who were familiar with EEA believed that the science was still too uncertain. In the laypeople’s focus groups, the divergent EEA results led many participants to revert to their prior beliefs that the role of climate change in the drought was secondary and to doubt that the EEA science could explain specific weather events. Policy makers also remained ambivalent about the usefulness of EEA.

These results suggest that while EEA holds interest for scientists, it is not yet widely accepted and applied by stakeholders nor more broadly by laypeople, and may even counterproductively reinforce views that climate change plays a minor role in most EWEs. This outcome reinforces the importance of understanding laypeople’s attributions for events. In addition, science communicators need to clarify that scientific knowledge evolves as new evidence is discovered and is not fixed like ideological beliefs. There is a clear analogy with the COVID-19 pandemic, where scientists have changed some of their messages in response to new findings. Laypeople may see these changes as showing a lack of consensus among scientists, as noted above, but scientists normally adjust their views with the emergence of new data, and see that responsiveness to new evidence as a fundamental strength of the scientific method (Lewis 2020).

## Laypeople’s attributions for events

Like scientists, laypeople explain actions and events in their lives; and these lay attributions are important for people to understand and predict their environment. Researchers in both psychology (Försterling 2001; Weiner 2018) and law (Hart and Honore 1985; Moore 2009) have sought to model these lay attributions for events and actions. Initially, classical psychological theories of attribution assumed laypeople are like scientists, and follow a more or less normative model of causal inferences. However, more recent models of lay attribution were developed to explain departures from such normative models. Although these models of lay attribution were developed independently of EEA science, they may provide guidance about ways to communicate EEA science to laypeople who lack scientific training. We do not present a full review of all research on laypeople’s attributions here, as this would fill a book (Försterling 2001; Weiner 2018); instead, our goal is more theoretical in describing some key aspects of lay attributions that we believe are relevant to EWEs. We also suggest how science communicators may use this knowledge to influence citizens’ attributions for EWEs. Rather than simply giving laypeople more information, which may overload or confuse them, these strategies entail communicating more precise messages that target lay attribution in specific ways. These strategies are summarised in Table 1 and elaborated in the sections below.

**Table 1:** Summary of citizens’ patterns of attributions for events, with suggested intervention strategies to influence citizens’ attributions for EWEs

	Citizens’ (lay) attributions	Strategies to influence citizens’ attributions for EWE
**1. Number of causes**	Prefer simple (single cause) explanations (Kelley 1973; Lombrozo 2007)	Spell out multiple factors in EEA; use analogies with events where multiple causes are more accepted: e.g. cancer, earthquake damage
**2a. Influence of covariation** (correlations between causes and events)	Often overlook covariation (Kelley 1967; Schwarz 2012)	Clarify covariation in lay terms: e.g. current climate change follows from increases in GHG emissions, not other causes
**2b. Influence of causal mechanisms** (events that lead to the given outcome)	Prefer mechanisms to covariation (Johnson and Ahn 2015; McClure et al. 2007b)	Describe mechanisms in lay terms: e.g. GHG emissions create a gas ‘blanket’ around the earth that prevents heat from escaping
**3. Counterfactual and sufficient causal effects**	Prefer sufficient causes and discount counterfactual probabilities (McClure et al. 2007a; Pearl 2009)	Clarify importance of counterfactuals: e.g. *X* event is unlikely to have happened without GHG emissions
**4. Mental models, narratives; controllability of causes**	Mental models omit key controllable causes (Bostrom et al. 2013; McClure 2017)	Review laypeople’s mental models; use narratives; affirm the role of controllable causes (e.g. GHG emissions) in climate change
**5. Influence of motivation and misinformation**	Motivation has strong effect on attributions (Cook 2019; Graham and Keller 2020)	Challenge misinformation and spell out source/motive: e.g. vested interests, desire to maintain current lifestyle

*EWE*, extreme weather event; *GHG*, greenhouse gases; *EEA*, extreme event attribution

### Multiple versus single causation: how many causes do laypeople infer?

The scientific understanding of EWEs recognises multiple causal influences. For example, NASEM (2016) suggests ‘many conditions must align to set up a particular’ extreme weather event (p. 28). Likewise, classical psychological theories of causal attribution proposed that laypeople use multiple causes to explain an extreme event, although they tend to invoke a single cause and discount other causes for an ordinary event (Kelley 1973; Wilkerson and Meyer 2019). For example, these models predict that laypeople may explain a student’s very high exam mark in terms of his or her ability *and* effort, though attribute a student’s average mark to a single cause of either the ability *or* effort (McClure 1998).

Although there is some support for the classical theory that laypeople invoke multiple causes to explain extreme events (Morris and Larrick 1995; Wilkerson and Meyer 2019), for many extreme events, research shows laypeople prefer a single compelling cause rather than multiple causes (McClure 1998). For example, to explain Einstein’s impressive achievements in science, laypeople do not say he was intelligent and worked hard and had good luck and so on; they just say he was a genius (McClure et al. 1991). This pattern is more common where questions use open-ended formats that let people say whatever they think rather than making causal ratings on structured scales. Other research similarly shows that laypeople often prefer simple attributions with few causes (Lombrozo 2007; Wilkerson and Meyer 2019) and differ from scientists’ attributions in overlooking multiple causal influences in events.

The same applies to extreme disasters. For example, when buildings collapse in earthquakes, scientists recognise that the damage is a result of multiple factors such as poor building design, the triggering plate movement at the fault-line, how the shock was propagated through the surrounding soils, and so on. In contrast, most laypeople attribute the damage simply to the earthquake motion and discount controllable factors such as building design (McClure 2017). A similar lay over-simplification of the chain of events is likely to apply to EWEs, where scientists recognise the roles of multiple natural and anthropogenic factors and assign weights to them (Lidskog and Sjödin 2015). In contrast, laypeople may prefer a single cause, possibly a cause to which the scientists assign a lesser weight, and one that is unrelated to climate change.

Rather than attributing an EWE to climate change, laypeople may choose other explanations (such as natural weather variability), especially causes proximal to the event (McClure 2017). To illustrate, a rare study on laypeople’s attributions for wildfire in the Sierra Nevada Mountains (USA) showed that laypeople mostly invoked proximal events close to the fire, such as lightning, arson, or forest management (Kumagai et al. 2004). They overlooked climate change, which is more of a background or distal factor. Participants who directly experienced the fires attributed the fires more to forest management whereas others attributed the fires more to actions such as discarded cigarettes. In neither case was anthropogenic climate change cited as a cause, an omission that may reflect the fact that laypeople focus on proximal causes (and possibly that participants were less aware of climate change at the time of this study).

In sum, laypeople’s preference for simple explanations and proximal causes that closely precede events may lead them to discount the role of anthropogenic climate change in EWEs. This observation is linked to the psychological distance of climate change, manifested in feelings that severe climate impacts are too uncertain, will occur far away, far in the future, and to people different from oneself (Milfont 2010; Spence et al. 2012). To address laypeople’s preference for single cause attributions and proximal causes, science communicators can spell out that EWEs reflect multiple factors and that attributions for these events that discount anthropogenic GHGs provide an incomplete account. Providing a well-accepted illustration of multiple causes may help. Take smoking and lung cancer as an example. It is nowadays clear to most people that smoking is associated with an increased risk of lung cancer, and equally that there are many other genetic, behavioural, and environmental factors that contribute to a person getting sick with this disease. The denialism movement that has eventually lost credence in this sphere (after many years of massive spending and disastrous delays) may provide a useful comparator to climate change (Oreskes and Conway 2010).

### Which influences shape lay attributions? 1: Covariation and causal mechanisms

As we noted, classical models of lay attribution proposed that citizens’ attributions use a logic similar to scientists’. Most notably, lay attributions have been said to reflect the covariation between possible causes and an event (Kelley 1967; Muschetto and Siegel 2021; Schwarz 2012). This idea implies that laypeople attribute events to those causes that are present when the effect occurs and absent when the effect does not occur. This model was construed in terms of attributions for human actions but can be extrapolated to physical events such as earthquakes (McClure 2017). When applied to climate change, this model suggests that the consistent covariation between anthropogenic GHG emissions and the subsequent rise in global temperatures across locations should lead laypeople to realise that the emissions are influencing the global temperatures. That other possible influences do not co-occur with global heating (i.e. the relationship to GHGs is distinctive) should enhance laypeople’s attribution of the heating to GHGs. Although this model has not been applied to lay attributions about EWEs, research on attributions in other spheres suggests that laypeople use the covariation principle when they are given the relevant causal information, such as the consistency between a potential cause and effect (see, e.g. Maris and Hoorens 2014).

Nevertheless, a potential issue about using the covariation principle in climate EWE communication is that the temperature increase may show a delay after the GHG emissions, and this delay is one of the things that makes climate change more difficult to comprehend for laypeople, who link causal effects to sequences with a short time lapse and to proximal causes (Michotte 1963; Moore 2009; Pawlik 1991). Also, since climate change (as high GHG levels in the atmosphere) is now always present, covariation with GHGs may have a weak effect on citizens’ perception of EWEs and they may not attribute these specific events to climate change. To make use of the covariation principle in climate change communication, scientists may need to spell out more clearly that EWEs increased in frequency in recent years and at the same time we have experienced an upswing in GHG emissions.

However, there is an additional issue. Laypeople may not use covariation systematically in their spontaneous judgments, and people often lack covariation data or simply ignore it, partly because they often need to make quick causal judgments (Schwarz 2012). In such cases, laypeople use single cause explanations as described in the previous section and prefer explanations that include a mechanism that explains why an effect occurs (Johnson and Ahn 2015; McClure et al. 2007b). Regarding climate change, scientists have well-developed theories that spell out mechanisms whereby GHG emissions contribute to EWEs. For example, global warming leads to ocean warming, which increases moisture in the atmosphere, which in turn leads to higher rainfall during powerful storms and hurricanes (Walsh et al. 2019). Laypeople who lack scientific knowledge may not comprehend these chains of mechanisms affecting such extreme events, so their determinations of attributions may not reflect this knowledge. However, laypeople can understand mechanisms that are clearly communicated, and they seek mechanisms that explain events (Johnson and Ahn 2015); so laypeople’s limited knowledge of the science may be surmounted by clear communications of these mechanisms using minimal jargon.

In sum, to engage laypeople’s understanding of covariation and mechanism information relating to EWEs, climate scientists and communicators can point out that recent extreme events covary with GHG emissions over a long time period, and more than other possible causal influences that laypeople may invoke, such as natural cycles.

However, since laypeople are more convinced by mechanisms than covariation information, scientists can link covariation to causal mechanisms and explain how the increase in GHG emissions traps gases in the atmosphere and creates conditions that explain patterns of EWEs. This account represents a more plausible explanation of the changed frequency and intensity of these events than natural influences on their own. Of course, many scientists are already communicating covariation and mechanism information about extreme events, but understanding how laypeople make attributions, particularly their preference for mechanisms, could sharpen these communications. Obviously, these attributions need to be phrased in jargon-free terms laypeople can understand.

### Which influences shape lay attributions? 2: Counterfactuals, probabilities, and sufficient causes

In addition to downplaying covariation between causes and effects, many laypeople do not realise that climate scientists’ claims about climate change invoke counterfactual probabilities (Hannart et al. 2016; NASEM 2016). Counterfactual reasoning is used because all weather events reflect multiple causal influences and counterfactual probabilities clarify how likely a given event is to have occurred in the absence of climate change—and GHGs (Pearl 2009; Roese and Epstude 2017). Climate scientists use counterfactual probabilities to point to the role of climate change in many EWEs. That is to say, the science of EEA says ‘had there been no climate change, this extreme weather event probably would not have happened or would have been less intense’ (e.g. see Otto et al. 2012). Climate scientists do not claim that climate change is a sufficient cause—that is, causes that are always followed by EWEs.

Counterfactual probabilities relate to necessary causes, which are causes without which an effect would not occur. Some climate scientists see necessary causes as a proxy for counterfactual probabilities in analyses of extreme events (Hannart et al. 2016), although other climate scientists avoid this causal phrasing (NASEM 2016). Hannart et al. (2016) claim some climate scientists do not spell out the probabilistic nature of their extreme event attributions, and laypeople mistakenly think scientists are implying climate change is a sufficient explanation of these events. In particular, Hannart et al. (2016) state that ‘it is important to distinguish between necessary and sufficient causality. Such a distinction is, at present, lacking in the conventional event attribution framework. Any time a causal statement is being made about a weather or climate-related event, part of the audience understands it in a necessary causation sense, while another part understands it in a sufficient causation sense, which can give rise to many potential misunderstandings’ (Hannart et al. 2016, p. 108).

This point is important because laypeople differ from scientists in focusing more on sufficient causes of events than probabilities or necessary causes (McClure et al. 2007a). Research demonstrating this point used scenarios where causal chains of intentional and physical events led to a harmful outcome (e.g. a youth setting fire to a shrub, followed by wind fanning the flames, leading to a forest fire; see Hilton et al. 2016; McClure et al. 2007a). The research examined whether laypeople’s preference for different attributions of outcomes reflected the counterfactual probability or sufficiency of the attributions for the outcome. Participants judged each attribution for its quality (e.g. ‘How good is the statement “a youth set fire to the shrub” as an explanation of the fire?’), its sufficiency (e.g. ‘How probable was it that the forest fire would occur after the youth set fire to the shrub?’), and its counterfactual probability (e.g. ‘How probable would the forest fire have been if the youth had not set fire to the shrub?’). Participants’ judgments of the quality of the causes correlated with the sufficiency more than the counterfactual probability of each cause, suggesting that citizens prefer sufficient causes over counterfactual factors. This focus contrasts with climate scientists’ focus on counterfactual probabilities to clarify the role of climate change in EWEs.

One reason why laypeople judge the sufficiency of a cause as more important is that they see the relevance of a cause to an outcome as a key criterion of causality (McClure et al. 2007a). To illustrate, the presence of oxygen increases the probability of a fire (and is necessary for fire); but laypeople do not see oxygen as relevant to explain any given fire—as oxygen is always present, they take it for granted—in contrast to events like dropping a cigarette (Mackie 1980). As noted above, since GHG emissions and climate change are now always present, just like oxygen, this feature of climate change may partially explain why laypeople may not easily attribute EWEs to climate change (see Harrington et al. 2022).

Even when laypeople do think in terms of probabilities, they judge probabilities differently than scientists. Climate scientists’ EEA involves identifying changes in (even small) probabilities due to anthropogenic climate change. However, laypeople find it challenging to account for small probabilities (Spiegelhalter 2019). The following example may clarify the issue. Floods are typically identified as 1 in 100-year events (most flood hazard maps use this arbitrary threshold to define ‘flood prone’). In making a scientific EEA, climate scientists may point to a shift of a specific flood likelihood from 1 in 100 years (1% probability), if there was no climate change, to 1 in 75 years (probability of about 1.3%) if there is climate change. This is a 30% increase in likelihood, a very significant attributable increase in risk, or a fraction of attributable risk of 0.25 for climate scientists. However, it may look almost immaterial to most laypeople. In two recent heatwaves, however, scientists have identified a much higher increase in probability: the Western North America heatwave in June 2021, and the UK heatwave in July 2022 (Philip et al. 2021; and Zacariah et al. 2022, respectively). For both, the research suggests that these events may have been ‘statistically impossible’ without climate change. It is possible that laypeople may judge these probabilities very differently, based on random salient information.

More broadly, laypeople often exhibit biased judgments of low probability risks, where salience determines these judgments rather than an informed assessment based on the existing scientific knowledge (e.g. people’s fear of sharks when compared to the relative absence of fear from driving cars). For example, those who experienced Hurricane Alicia in Houston in 1983 may have generalised their salient experience of Alicia, which was not linked by scientists to anthropogenic climate change, to the case of Hurricane Harvey in Houston in 2017, thereby erroneously concluding that Harvey is not linked to climate change, as well.

In sum, concerning different causal influences, science communicators can clarify the importance of climate change as a counterfactual factor in extreme events, in that these events are less likely to have occurred in the absence of climate change. Climate scientists could communicate their knowledge on counterfactual and probable causes of these events in ways that take account of laypeople’s tendency to think in terms of sufficient causes. For example, laypeople may think that because natural processes contribute to extreme events, these processes are sufficient to explain those events. Science communicators could be explicit about counterfactuals (Hannart et al. 2016) and say ‘We might not have suffered from this extreme weather had we not caused climate change’. Research shows that most citizens do understand the concept of counterfactuals, especially after a personal loss (‘If only we hadn’t….’; Roese and Epstude 2017), even if they use different terms to those used by scientists.

### Attributions in context: the role of mental models and narratives

Attributions for events do not occur in a conceptual vacuum. Bostrom et al. (2012) showed that laypeople’s attributions for events reflect their mental models about these events. Laypeople’s mental models of climate change may shape their attributions and their support for actions to mitigate climate change. For example, those who ascribe climate change to natural causes (such as natural long cycles) favour geo-engineering solutions, whereas those who see GHG emissions as the main factor support policies reducing emissions (Bostrom et al. 2012).

In a similar vein, laypeople tend to construct their worldview around narratives (Johnson and Ahn 2015). Shiller (2017) uses the idea of narrative to mean ‘a simple story or easily expressed explanation of events that many people want to bring up in conversation or on news or social media because it can be used to stimulate the concerns or emotions of others, and/or because it appears to advance self-interest’ (p. 968). These narratives spread and often have important consequences, not only for beliefs, but also for the actions that follow from these beliefs. In Shiller’s framing, exogenous events (like a recession) lead to the formulation of these narratives (e.g. a narrative emphasising the importance of thrift). Once the narrative is created, it can spread like an infectious disease. In the case of EWEs, a single occurrence of a catastrophic event, say a large wildfire, may lead to the creation and propagation of a narrative around the causes of that wildfire. These narratives may take the form of conspiracy theories of climate change denial and then spill into laypeople’s attributions for specific weather events (Haltinner and Sarathchandra 2021).

Mental models and narratives may highlight either controllable or uncontrollable causes. When people attribute events to uncontrollable causes, this inference increases their fatalism about the potential for reducing negative events (Aitken et al. 2011; Weiner 2018). In contrast, when laypeople attribute events to controllable causes, they infer that the events can be prevented and that actions to mitigate the events are worth taking (e.g. McClure 2017; Weiner 2018). Laypeople whose causal models recognise that the anthropogenic sources of EWEs are controllable support mitigation policies more than people who cite only uncontrollable factors (Bostrom et al. 2013).

Notably, it is possible to change people’s attributions to more controllable causes. For example, in strategies for countering lack of motivation, psychologists work to change clients’ attributions for negative events from uncontrollable causes (‘It’s impossible to change’) to more controllable causes (‘I need to work harder on it’), as this shift is effective in enhancing motivation (e.g. Seligman et al. 2013; Weiner 2018). Psychologists do this by (gently but consistently) challenging the person’s misleading attributions. Similarly, scientific communication about EWEs can include strategies to enhance attributions to controllable GHGs in the chain of events and challenge fatalistic attributions that discount these factors (Bostrom et al. 2013). This strategy may increase people’s action to mitigate climate change (Aitken et al. 2011; Lidskog and Sjödin 2015).

More broadly, in regard to mental models and narratives, climate science communicators can apply the ‘storyline’ approach to extreme events which examines the role of each factor contributing to each event as it unfolded, as in an accident investigation (Shepherd 2016). This storyline approach is closer to the way laypeople perceive risk in terms of narratives than a probabilistic account (Shepherd et al. 2018), and so is more likely to influence citizens who in turn influence policy makers (Jezequel et al. 2018). Climate science communicators can thus present a storyline narrative in which their attributions for extreme events are embedded, as a way to tap into laypeople’s way of attributing events.

### Motivated biases in citizens’ attributions

Attribution research shows that while laypeople’s attributions for events partially reflect cognitive and logical processes, they also reflect motivated reasoning that is very different to scientific reasoning (Weiner 2018). As a result, laypeople often generate very different attributions to scientists—in many domains. As in these other domains (e.g. COVID-19), laypeople may dismiss scientists’ claims about the role of GHGs in weather events and claim that these events are wholly due to other causes such as natural variability in the weather, or random human actions such as arson or flawed forest management (Cook 2019). These lay attributions are fuelled by misinformation such as people claiming that the 2020 Australian bush fires were primarily due to arson, not climate change. This misinformation can be exacerbated by bots and trolls on the internet, which further undermine laypeople’s willingness to engage with climate change issues (Graham and Keller 2020). Rather than drawing on overall global trends in weather patterns to assess the role of climate change, some laypeople cite random instances of cold weather as proof that climate change is not happening (Cook 2019). Thus, some laypeople cherry-pick exceptions and dispute overall trends (Weber 2016).

Laypeople’s attributions for weather events are subject to diverse motivations. Research on motivated cognition shows that laypeople process information to fit conclusions that suit some motive (Braver et al. 2014). Many actions, such as eating meat and driving petrol cars, generate GHG emissions that contribute to climate change; and changing these practices requires major changes to people’s lifestyles (Gifford 2011). This challenge provides strong motivation for laypeople to dismiss the idea that human actions such as their own contribute to climate change and EWEs. Research shows that laypeople in the USA who choose to live in coastal locations exposed to sea-level rise are more likely to discount climate change (Bernstein et al. 2021; but see Brody et al. 2007; Milfont et al. 2014). More interestingly, people who live in coasts that have experienced more sea-level rise are also *less* likely to believe that the seas are likely to rise more in the near future (Shao et al. 2020). This bias reflects their motivation to deny the risk. By acknowledging the role of anthropogenic climate change, people face the realisation that they would incur costs (either monetary costs, or well-being costs related to changing their preferred habits). This gives laypeople a strong incentive to believe otherwise, and to attribute EWEs to some other cause—one that does not entail a significant mitigating cost.

At the extreme end of this, the writer Upton Sinclair noted that ‘It is difficult to get a man (sic) to understand something, when his salary depends on his not understanding it’ (as cited in Ratcliffe 2016). This is also the case with climate change. Many vocal climate denialists’ voices have been funded by the fossil fuel industry (Cook 2019). These agents challenge the consensus among climate scientists on the role of human actions in EWEs and climate change in general (Cook 2019), and steer many laypeople away from reliable scientific evidence on climate change.

To counter these motives and incentives affecting lay attributions, science communicators can more clearly identify these motivations that lead some to discount the role of climate change in EWEs in spite of the scientific explanation of this role, and explicitly point to the interests of funders (Cook 2019). Climate science communications could spell out specific examples of the source of misinformation and the motivation behind it.

## The broader context of climate change

While the science of attributions for specific weather events is burgeoning, climate scientists can further re-affirm that the evidence for anthropogenic factors in climate change is beyond scientific dispute and is amply reflected in trends such as sea-level rise. Some climate scientists argue that given the pervasive effects of anthropogenic factors on the climate, it makes little sense to talk about whether a single event was affected by anthropogenic influence (NASEM 2016). However, scientific attribution for extreme events provides ‘an explicit connection between climate science… and the specific event in the news, making the science concrete in a way that statements about broader trends and future projections do not’ (NASEM 2016, p. 19). Strategies to modify citizens’ attributions for specific weather events may therefore also reduce people’s scepticism about the anthropogenic cause of the changing climate more generally.

In addition, extreme weather event attributions in the context of a changing climate are required for the risk calculations that affect many public policies, such as building codes, planning, and insurance policies (NASEM 2016; Pastor-Paz et al. 2020; Stott et al. 2016). There is a need to communicate these attributions to the public in ways that take account of the processes behind lay attributions. To be effective, these risk communications need to translate technical concepts and probabilities into terms understood by most laypeople (Kumagai et al. 2004; Moser 2010; Shepherd et al. 2018). These communications can draw on research showing which types of extreme events affect laypeople’s attention to climate change (Sisco et al. 2017).

## Conclusions

This paper describes factors that have been shown to influence laypeople’s attributions for events. It then spells out how this understanding might enable climate scientists to communicate the science of EEA more effectively by tapping into the way laypeople infer causes for events. This also applies to science media who play a key role in communicating science to the general public (Moser 2016). This paper has introduced the topic and suggested some key relevant concepts and strategies. To highlight the main points, we suggest that laypeople may typically say EWEs have always happened and are caused by ‘natural’ processes or accidents like lightning/arson and there is nothing we can do to stop it (uncontrollable causes, proximal causes, simple causal structure). In response, we suggest that scientific communications about an extreme weather event can tell (1) a clear *narrative* that highlights climate change as one of the *multiple necessary causal influences*, including the *counterfactual* that this particular extreme event ‘is less likely to have occurred or been so intense without climate change’, while (2) describing *causal mechanisms* of anthropogenic climate change, emphasising the relative *controllability* of GHG emissions and (3) pointing to *covariation*—EWEs have increased in frequency and intensity as GHG emissions have increased over time. These factors need to be considered against the role of motivated resistance as a barrier to effective communication and action.

There is more that might be done to clarify citizens’ attributions in relation to EWEs. Of course, the strategies suggested here to address laypeople’s attributions for extreme events will not be sufficient on their own to change people’s perceptions of these events. However, these strategies can be combined with other methods which have been shown to be effective in countering misinformation about climate change (Meertens et al. 2021). Examples are messages that frame risks in ways that are compelling to the public and that draw on the co-benefits of actions that mitigate climate change. These strategies, in turn, may be even more effective if they address the attribution reasoning that leads laypeople to discount the role of climate change in extreme weather events and other consequences of climate change.
